# Robot Assisted MRI-Guided LITT of the Anterior, Lateral, and Medial Temporal Lobe for Temporal Lobe Epilepsy

**DOI:** 10.3389/fneur.2020.572334

**Published:** 2020-11-27

**Authors:** Kunal Gupta, Adam S. Dickey, Ranliang Hu, Edward Faught, Jon T. Willie

**Affiliations:** ^1^Department of Neurosurgery, Emory University Hospital, Atlanta, GA, United States; ^2^Department of Neurosurgery, Indiana University Health, Indianapolis, IN, United States; ^3^Department of Neurology, Emory University Hospital, Atlanta, GA, United States; ^4^Department of Radiology, Emory University Hospital, Atlanta, GA, United States; ^5^Department of Neurological Surgery, Washington University School of Medicine, Washington, DC, United States

**Keywords:** temporal lobe epilepsy, LITT (laser interstitial thermal therapy), ROSA (robotized stereotactic assistant), temporal lobectomy, SEEG (stereoelectroencephalography)

## Abstract

Robotic systems have fundamentally altered the landscape of functional neurosurgery. These allow automated stereotaxy with high accuracy and reliability, and are rapidly becoming a mainstay in stereotactic surgeries such as deep brain stimulation (DBS), stereoelectroencephalography (SEEG), and stereotactic laser ablation/MRI guided laser interstitial thermal therapy (MRgLITT). Robotic systems have been effectively applied to create a minimally invasive approach for diagnostics and therapeutics in the treatment of epilepsy, utilizing robots for expeditious and accurate stereotaxy for SEEG and MRgLITT. MRgLITT has been shown to approach open surgical techniques in efficacy of seizure control while minimizing collateral injury. We describe the use of robot assisted MRgLITT for a minimally invasive laser anterior temporal lobotomy, describing the approach and potential pitfalls. Goals of MRgLITT are complete ablation of the epileptogenic zone and avoiding injury to uninvolved structures. In the middle fossa these include structures such as cranial nerves in the skull base and cavernous sinus and the thalamus. These can be mitigated with careful trajectory planning and control of laser ablation intensity.

## Introduction

Advancements in robotic and laser technology have dramatically altered the landscape of functional neurosurgery. The principal application of robotics in functional neurosurgery has been automated stereotaxy, providing a potentially expeditious workflow for cases involving multiple sequential and unrelated trajectories, as exemplified by stereoelectroencephalography (SEEG). A number of robotic stereotactic devices are currently available in North America, including the Neuromate robotic system (Renishaw), ROSA ONE Brain robotic platform (Zimmer Biomet), and Stealth Autoguide cranial robotic guidance platform (Medtronic). Robots are rapidly becoming a mainstay in surgical stereotaxy and studies have demonstrated that the accuracy of robotic guidance systems can approach that of gold-standard stereotactic frames (1–4).

Likewise, the modern surgical application of laser technology combines a number of advancements, narrow-caliber cooled-fibers for laser interstitial thermal therapy and magnetic resonance thermography for non-invasive real-time imaging of tissue temperature, into a minimally invasive therapeutic strategy. Two commercial platforms utilizing this technology are currently available for central nervous system application in North America, NeuroBlate (Monteris) and Visualase (Medtronic). Robotic stereotaxy and laser technology can be combined as a minimally invasive therapy, providing surgeons a method to ablate tissue across a number of subspecialties including epilepsy and neuro-oncology.

Laser ablation of the temporal lobe is especially challenging due to its complex shape and volume. MRgLITT has been successfully applied to ablation of the mesial temporal lobe for laser amygdalohippocampotomy, with up to 53% freedom from focal seizures impairing awareness reported after 12 months (5, 6), with variable outcomes likely resulting from important differences in patient selection and technical execution. In particular, temporal lobe epileptogenic zones may extend outside the entorhinal cortex, amygdala, and hippocampus. However, previous case series of MRgLITT for temporal lobe epilepsy describe approaches intended to deliver a minimally invasive analog to an open selective amygdalohippocampectomy. In cases where a wider epileptogenic zone is identified by SEEG or other approaches, a more extensive resection or ablation may be indicated. Complete laser ablation of the mesial, anterior and lateral structures requires a combination of multiple lateral and posterior approaches, which could be difficult to achieve expeditiously with a stereotactic frame set up in a single configuration. Advances in robotic stereotactic targeting allows the placement of multiple LITT bolts and laser cannulae expeditiously and accurately, with potentially fewer restrictions upon available trajectories (7, 8). Here, we present the use of robotic assisted MRgLITT for ablation of the anterior temporal lobe using multiple stereotactic trajectories to create a temporal lobe ablation volume analogous to open anterior temporal lobotomy.

## Case Report

A 32-year-old right handed female presented with a 2-year history of medically refractory right temporal lobe epilepsy. Her seizures comprised 2 semiologies; the first consisted of focal seizures with déjà vu, out of body sensation and dream-like state, shortness of breath, nausea, diaphoresis, and bilateral hand paresthesia. These sometimes progress to behavioral arrest and loss of awareness. She reported post-ictal tiredness, fear and confusion. These initially occurred 2–20 times per day, however the frequency reduced to 1–3 times per month with lacosamide treatment, and followed a catamenial pattern. Her second seizure semiology consisted of generalized tonic seizures, characterized by arm extension and stiffening, lasting a few minutes, with 2 h of post-ictal confusion. These were infrequent, having occurred twice since the onset of her seizures. She has an existing diagnosis of depression and took citalopram for this. She denied specific risk factors, precipitating events, or other psychiatric comorbidities. She reported, however, that she was hospitalized with fever of unknown origin at age 19-years-old and again at 20-years-old (10–11 years prior to seizure onset). Her other medical history was unremarkable and she had not had prior epilepsy surgery or evaluation. She had failed trials of levetiracetam, oxcarbazepine, and lamotrigine. Cerebrospinal fluid and blood serology were negative for auto-antibodies. She was neurologically non-focal on examination.

Long-term video scalp electroencephalography (LTvEEG) captured 10 events. Two events were characterized by whole body tremors and pelvic thrusting without electrographic correlate (and were deemed non-epileptic events). For two events she reported a dreamlike state that often occurred prior to her seizures, however these had no electrographic change. Six typical events were captured, during which she described her typical semiology of a rushing feeling, bilateral hand paresthesia and nausea; she did not exhibit behavioral arrest. During these typical events, right frontal temporal polymorphic delta and theta activity were noted, maximal in F8/T2. She underwent neuropsychological testing, which demonstrated average to above average function with mild dysfunction in both verbal and non-verbal domains. While there was a slight split favoring non-verbal performance, other areas conflicted this, including better visual naming vs. auditory naming. Therefore, her neuropsychological testing was felt to be non-lateralizing.

**Figure 1:**
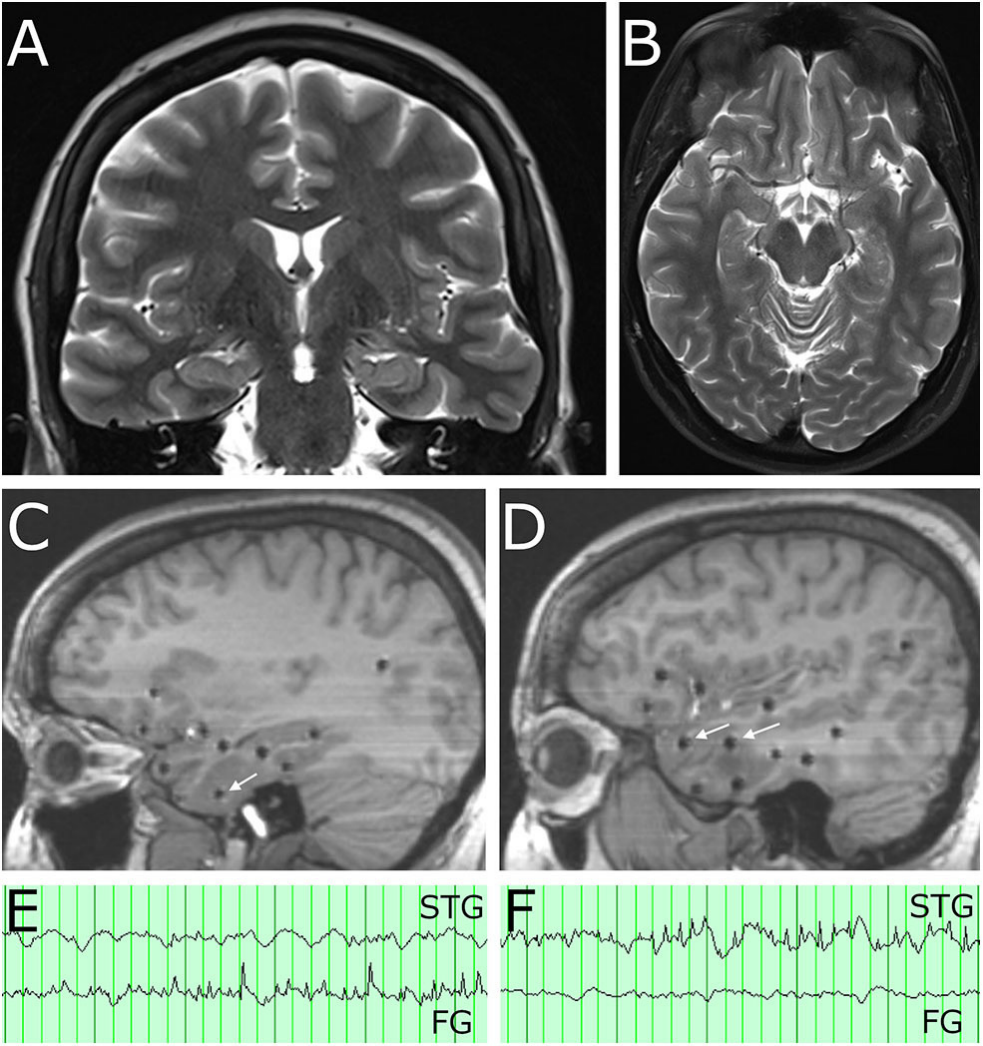
Pre-operative imaging demonstrates no abnormality on MRI and location of SEEG electrode contacts involved in the seizure network. **(A)** Coronal T2 MRI and **(B)** axial T2 MRI show normal appearance of the temporal lobes bilaterally. **(C)** Sagittal T1 MRI at the level of the mesial temporal lobe. White arrow indicates SEEG electrode contact in the fusiform gyrus with spread pattern electrographic activity during a captured seizure. **(D)** Sagittal T1 MRI at level of the lateral temporal neocortex. White arrows indicate SEEG electrode contacts in the superior temporal gyrus (anterior arrow) and middle temporal gyrus (posterior arrow) involved in spread patterns during subclinical seizures. **(E)** A clinical seizure (characterized by behavioral arrest and oral automatism) had EEG onset with rhythmic theta spiking, maximal on a contact in fusiform gyrus (FG) near the basal temporal pole, which evolved in frequency and morphology and then spread to other contacts in the neocortex of the superior, middle, and inferior temporal gyrus. Note that at onset of this clinical seizure, a contact in the superior temporal gyrus (STG) was not involved. **(F)** A subclinical seizure recorded on the same day had an onset of rhythmic theta spiking, maximal on a contact in the superior temporal gyrus (STG) which evolved in frequency and morphology and also spread to other contacts in the neocortex of the superior, middle, and inferior temporal gyrus. Note that at the onset of this subclinical seizures a contact in the fusiform gyrus (FG) implicated as the clinical seizure onset was not involved.

Her brain MRI showed no structural abnormalities (Figures 1A,B). Volumetric quantification of her hippocampi (NeuroQuant) demonstrated that her combined hippocampal volume was in the 95th percentile (9.41cc). Functional MRI demonstrated bilateral language lateralization (left > right Broca's area representation, left Wernicke's area, and right supplementary motor area activation) as well as bilateral parahippocampal gyrus activation with memory tasks. Positron emission tomography (PET) was performed; an area in the right Rolandic operculum demonstrated hypometabolism (*z* = −2.78) however this was felt to be discordant with her semiology and therefore of low clinical relevance during epilepsy multi-disciplinary team discussion. Ictal single-photon emission computerized tomography (SPECT) was attempted but injection of isotope was unable to be timed appropriately with a seizure. Results were therefore reported as interictal SPECT, which showed an area of hypometabolism in right inferior temporal lobe. Magnetoencephalography (MEG) demonstrated a single interictal spike in right medial basal temporal lobe. Her case was discussed in an epilepsy multi-disciplinary team meeting; given her semiology and LTvEEG findings it was reasoned that the most likely location for seizure onset was her right mesial temporal lobe. As she did not have mesial temporal sclerosis (and therefore did not meet criteria to skip invasive monitoring), it was recommended that she undergo stereoelectroencephalography (SEEG). Given mixed language dominance and potentially discordant neuropsychological memory results, she also underwent Wada testing, which unambiguously confirmed left sided support of memory and language.

Stereoelectroencephalography (SEEG) was performed targeting the right temporal lobe and related networks. Sampled locations included mesial structures (entorhinal cortex, parahippocampal gyrus, amygdala, hippocampus), basal structures (fusiform gyrus), lateral structures (superior, middle, and inferior temporal gyrus), and limbic lobe associated structures (insula, frontal and temporal opercula, orbitofrontal cortex, retrosplenial cingulate cortex). Twelve electrode arrays were placed in total, utilizing robotic assisted stereotaxy (ROSA robot), and the patient was monitored for 4-weeks. She had one typical clinical seizure (characterized by behavioral arrest and oral automatism noticed by patient's spouse) which was first detected in the contacts located in the fusiform gyrus (Figures 1C,E) with spread to the superior, middle, and inferior temporal gyrus. She also had subclinical seizures with a similar onset, as well as two independent seizure onset zones in the lateral superior temporal gyrus or the lateral inferior temporal gyrus (Figures 1D,F). In each of these three locations, however, low voltage fast activity was not detected, suggesting that the true epileptogenic zone had not been captured. Given the widespread and multi-focal right temporal involvement for the electrographic seizures, it was determined that the patient would benefit from anterior temporal lobectomy. Options of surgical therapy by conventional open anterior lobectomy vs. MRI-guided laser interstitial thermal therapy (MRgLITT) were presented, and she ultimately expressed a preference for a minimally invasive approach.

**Figure 2:**
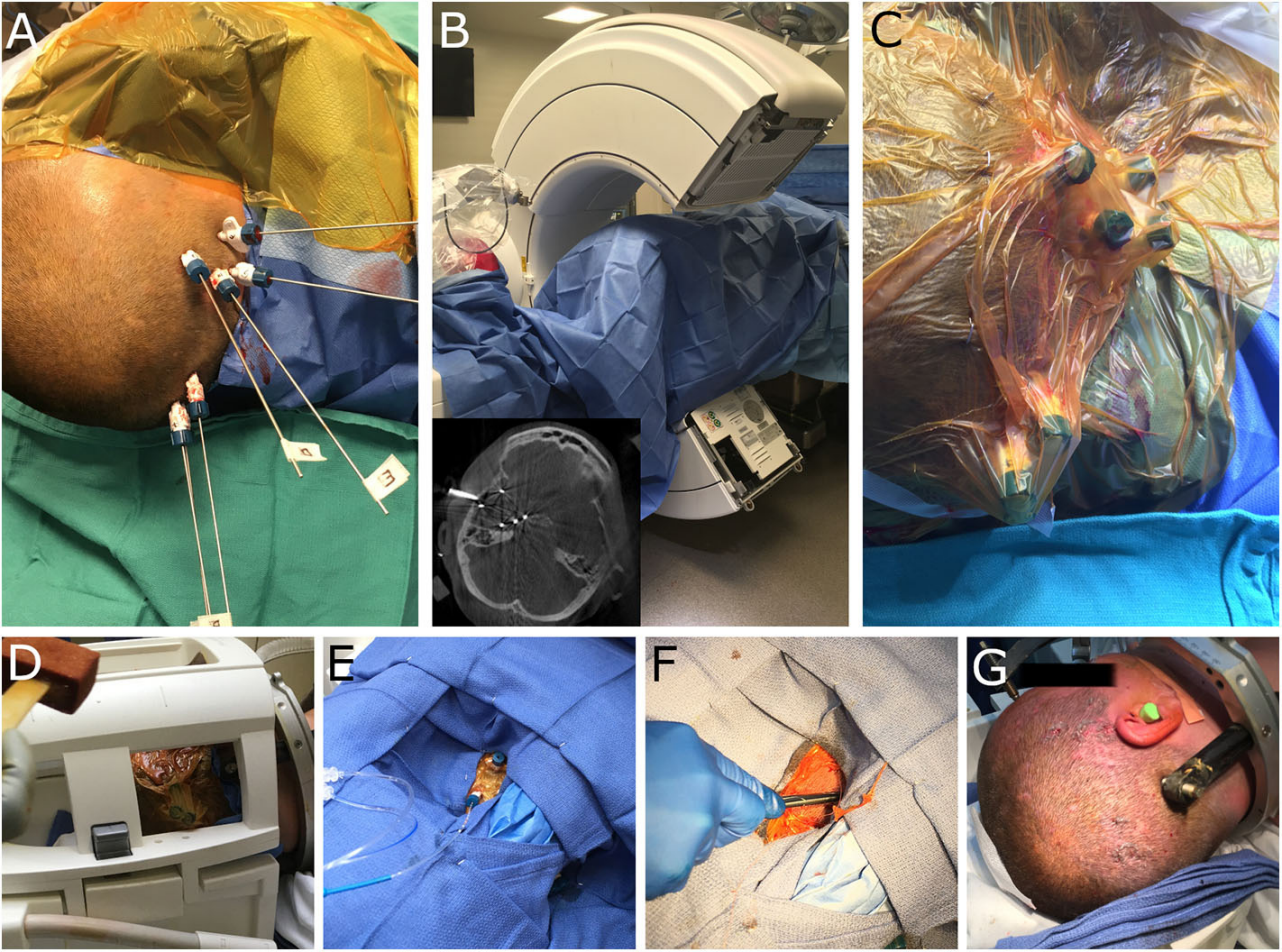
Intra-operative images demonstrating robotic workflow for implantation of laser bolts and subsequent MRgLITT in an interventional MRI suite. **(A)** Visualase bolts have been implanted and alignment stylets have been passed to target. **(B)** The patient is draped and the O-arm (Medtronic) is used to visualize each trajectory; inset demonstrates representative axial image and alignment stylets. **(C)** A sterile adhesive barrier (Ioban) is placed over the bolts prior to patient transfer to iMRI. **(D)** The head coil is placed and the area prepped with betadine. **(E)** Sterile towels are used to drape the head coil and allow access to the laser bolts; laser fibers are inserted through each bolt for laser ablation. **(F)** At the end of the case the bolts are removed and a single suture placed at each insertion site. **(G)** The end cosmetic result from multiple small incisions. Minimal hair removal is possible, however, in this case the patient shaved her own head prior to surgery.

MRgLITT was performed utilizing the ROSA stereotactic robot, at 6 trajectories encompassing the right temporal lobe. The patient's head was secured within a stereotactic frame base ring (CRW, Integra) and then affixed to the ROSA robot. Stereotactic registration was performed and the robotic articulated arm was navigated to each trajectory. In each location a twist-drill hole was made and laser bolts (Visualase, Medtronic) were placed. Once the bolts had been placed, alignment stylets were inserted to target (Figure 2A) and an O-arm (Medtronic) 3-dimensional image was obtained to ensure that the trajectories were accurate (Figure 2B). The distance to target from the top of each bolt was recorded for laser fiber insertion. The alignment stylets were removed and the bolts and surrounding scalp were covered with a sterile impermeable adhesive barrier (Ioban) (Figure 2C). The patient was transferred to the interventional MRI suite, positioned supine on the MRI table with the right shoulder bumped and the head turned laterally. A head coil was positioned to allow access to all the bolts and the adhesive barrier was prepped with betadine (Figure 2D). The area was then draped with sterile towels (Figure 2E) and the Ioban was removed for each trajectory, exposing the underlying sterile field. For each trajectory, and the laser fiber was inserted to the appropriate depth (Figure 2F), using the distance from the bolt to the target that had been recorded earlier.

**Figure 3:**
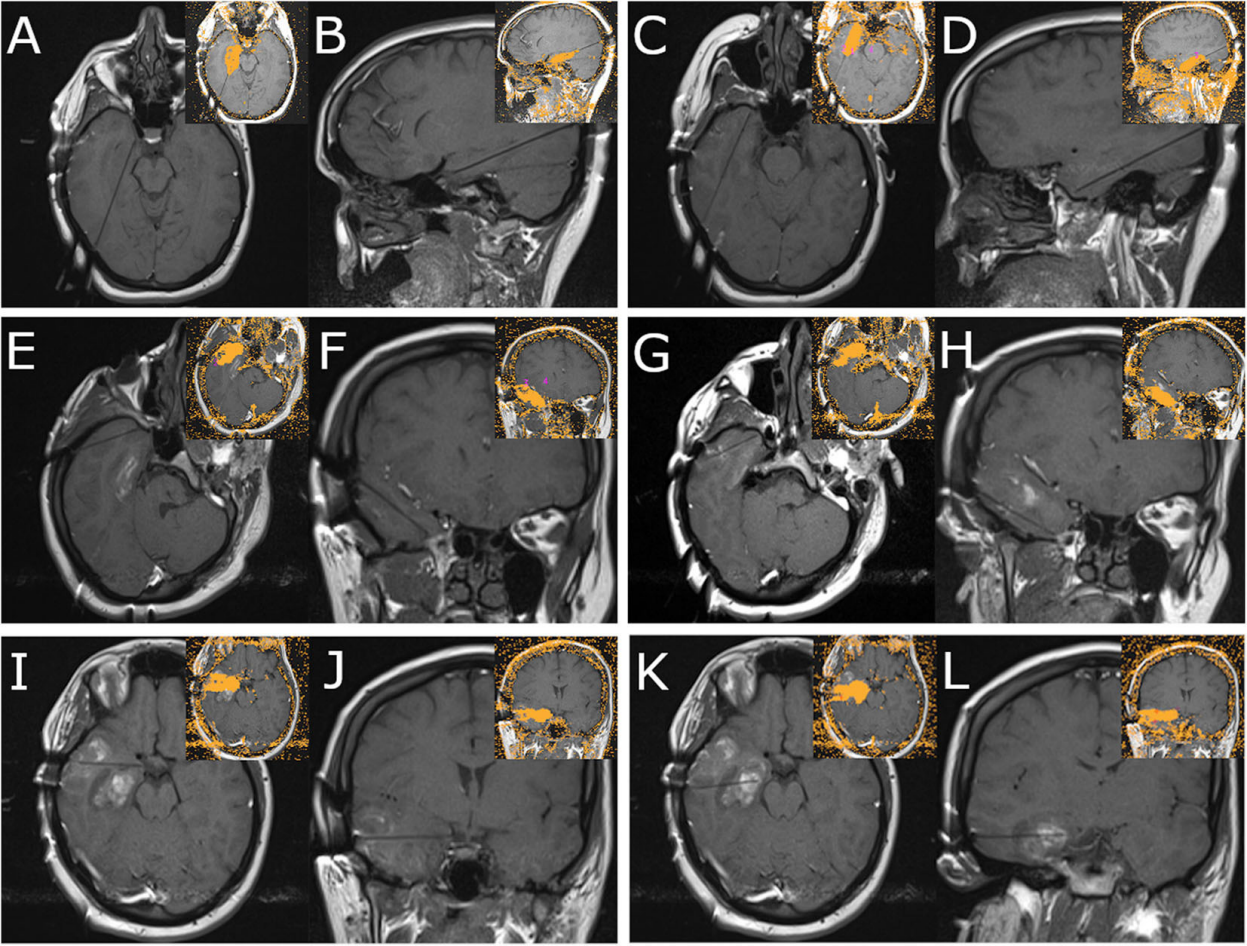
Panel demonstrates MRI images obtained during MRgLITT ablation of the right temporal lobe. Each panel demonstrates two orthogonal views along the laser fiber; inset images demonstrate the resultant ablation volume as determined by the Visualase system for each trajectory (Medtronic). **(A,B)** Panel demonstrates mesial hippocampal laser fiber trajectory. **(C,D)** Panel demonstrates medial temporal pole laser fiber trajectory. **(E,F)** Panel demonstrates superior lateral temporal pole laser fiber trajectory. **(G,H)** Panel demonstrates inferior lateral temporal pole laser fiber trajectory. **(I,J)** Panel demonstrates lateral approach to uncus laser fiber trajectory. **(K,L)** Panel demonstrates lateral approach to hippocampal body laser fiber trajectory.

**Figure 4:**
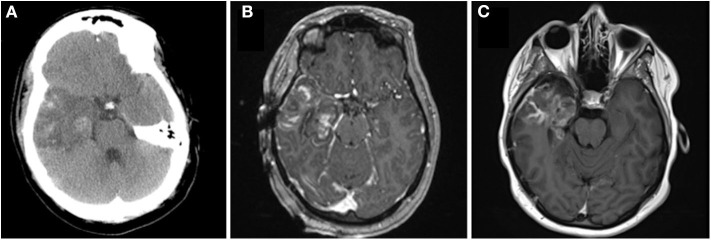
Panel demonstrates intra-operative and peri-operative patient imaging. **(A)** Axial CT obtained on post-operative day 2 demonstrates cerebral edema at the ablation site. **(B)** Post-ablation contrast-enhanced T1 axial MRI that demonstrates ablation of temporal lobe from the temporal pole to the level of the lateral mesencephalic sulcus and collicular plate at the time of surgery. **(C)** Contrast-enhanced (fluid dark) T1 axial MRI demonstrates the ablation 3 months after surgery.

A 980 nm/15 w diode laser (Visualase, Medtronic) was used to ablate all six trajectories, with the intention of confluent ablation of the medial temporal structures (extending posteriorly to the landmark of the lateral mesencephalic sulcus), as well as temporal pole, basal temporal lobe, and lateral temporal lobe extending 5 cm from the temporal tip. Figure 3 demonstrates orthogonal images for each laser fiber and the resultant Visualase ablation volumes for each trajectory. Two trajectories began in the parietal-occipital region to cannulate the long axes of the hippocampus/uncus (Figures 3A,B) and the rhinal cortices and medial temporal pole (Figures 3C,D), two oblique lateral trajectories began in the posterolateral temporal region and terminated in the superior (Figures 3E,F) and inferior (Figures 3G,H) lateral temporal pole, and two lateral trajectories completed the lateral neocortical ablation at the level of the uncus (Figures 3I,J) and the hippocampal body (Figures 3K,L). A final volumetric MRI confirmed the extent of the ablation (Figure 4B), and demonstrates the complete ablation of the targeted structures. The final ablation volume was 49.9 cm^3^ [itksnap.org (9)]. At the end of the case, the bolts were removed and a single interrupted suture was placed at each bolt site (Figure 2G). Total anesthesia time was 9.8 h, and total operative time was 8.85 h.

**Figure 5:**
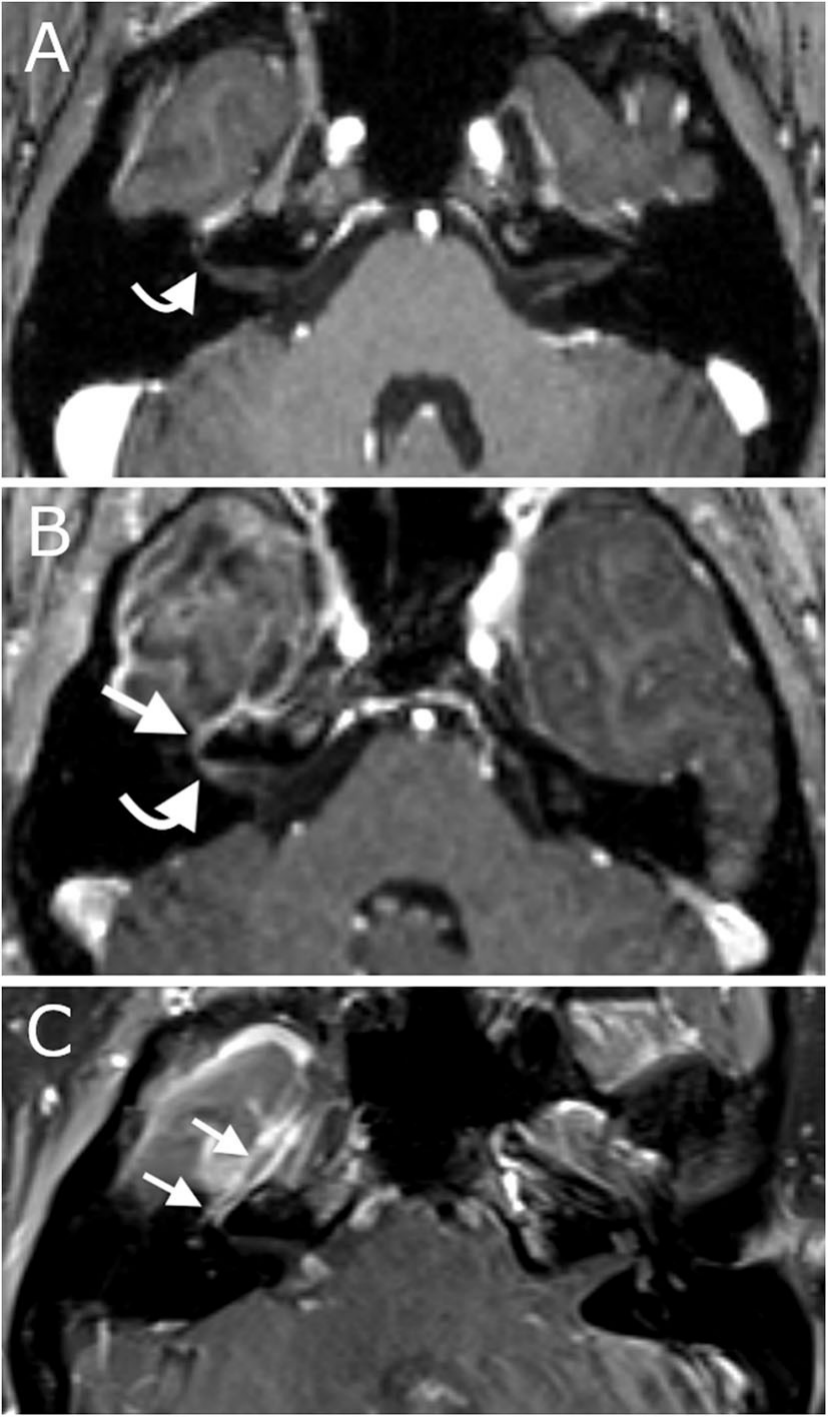
Panel demonstrates MRI scans of the internal acoustic meatus and facial nerve ipsilateral to the ablation. **(A)** Axial contrast-enhanced T1 pre-operative MRI demonstrates normal appearance of the facial nerve without abnormal enhancement. **(B)** Axial contrast-enhanced T1 intra-operative MRI demonstrates mild enhancement of the distal canalicular, labyrinthine and geniculate segments of the facial nerve at the time of surgery. **(C)** Coronal contrast-enhanced T1 MRI demonstrates intense enhancement of the facial nerve 3-months after surgery.

Immediately after surgery the patient had no gross neurological deficits. By post-operative day 1, however, she was noted to have the onset of right facial weakness. This gradually progressed by post-operative day 3 to an inability to close her right eye (House Brackman grade 4). Direct thermal injury during the ablation was considered unlikely to have occurred as the laser fiber was intentionally placed 8 mm away from the middle fossa skull base, and deficit took time to develop. She underwent repeat CT that demonstrated stable post-ablation changes (Figure 4A). She remained admitted for 3 days to ensure that radiographic imaging and clinical symptoms were stable prior to discharge. She was advised to tape the right eye closed to prevent exposure keratopathy. She was evaluated by ophthalmology as an out-patient: a left superior quadrantanopsia was noted on formal Goldman visual field testing; 3rd, 4th and 6th cranial nerves were functioning normally; and she was advised on continued eye-care to prevent exposure keratopathy. She and her husband reported cluster of 11 focal impaired-awareness seizures immediately after discharge however no further seizures by 6-weeks after surgery. At 6-weeks post-op she was noted to have worsening hemifacial weakness (now House Brackman grade 5). A brain MRI was obtained as an out-patient to evaluate her ablation (Figure 4C) and determine an etiology for her symptoms. Immediately after surgery there was evidence of mild enhancement of the distal canalicular, labyrinthine, geniculate and tympanic segments of the facial nerve (Figure 5B) compared to pre-operative imaging (Figure 5A). At 3-month follow-up there was intense perineural enhancement of the greater-superficial petrosal nerve, geniculate ganglion and tympanic segment of facial nerve (Figure 5C).

At 6-months after surgery, her hemifacial weakness had improved considerably to House Brackman grade 2, not visible at rest and she was able to close her right eye completely, and some residual reduced acuity of hearing with the right ear. She reported subjective headaches and insomnia but she did not find these symptoms bothersome enough to warrant further investigation. Her husband also reported multiple episodes of brief staring and unresponsiveness, and the patient denied recollection of these events, and given history of non-epileptic events, the episodes remained unconfirmed. These events occurred several times per week, without conversion to generalized seizure. Given the history of non-epileptic events, these episodes could not be discriminated from possible focal impaired awareness seizures, and lacosamide dosing was increased by her treating neurologist.

At 12-months after surgery, she reported complete resolution of her hemifacial weakness, however she acknowledged subjectively reduced acuity of right-sided hearing. She was referred to otorhinolaryngology, but chose not to pursue this. She denied new memory complaints and did not submit to post-operative neuropsychological assessment. Notably, follow up history and physical examinations did not detect visual defects or complaints, although we did not pursue formal ophthalmological evaluations. She and her husband denied any further episodes of impaired awareness since the increase in lacosamide dosing and no generalized seizures since surgery.

## Discussion

Robot-assisted MRI-guided laser interstitial thermal therapy provides a novel tool in the neurosurgeon's armamentarium for the treatment of epilepsy (and other subspecialties such as neuro-oncology and movement disorders). In particular, the surgical techniques that we describe combine novel robotic stereotaxy with laser technology to provide a minimally invasive approach for laser temporal lobotomy. A surgical strategy that utilizes robot-assisted stereoencephalography (SEEG) and MRgLITT is particularly powerful as it combines minimally invasive intracranial diagnostics with minimally invasive therapy. LITT ablation of the mesial temporal lobe has now been reported from multiple epilepsy surgery centers in North America, and such ablations have often utilized robotic and SEEG techniques (6). Nonetheless, a similarly large experience describing the potential indications, outcomes, and adversities of temporal lobe ablations that are large, complex, atypical, and/or extra-mesial remains to be established. This report stands out as an illustration of bringing multiple technologies to bear on a particularly atypical case and raises issues needing to be addressed in larger future series.

For patients diagnosed with MTLE, larger temporal lobe resections are associated with a greater chance of seizure freedom, presumably by eliminating occult epileptogenic tissues. The highly selective laser technique that is typically reported targets the amygdala, hippocampus, subiculum, and part of the entorhinal cortex. By contrast, open anterior temporal lobectomy generally also includes resection of the temporal pole, all of the rhinal cortices within the anterior fusiform and parahippocampal gyri, as well as anterior portions of the lateral temporal lobe, all of which may harbor occult epileptogenic tissues. Middle ground is represented by open “selective” amygdalohippocampectomy techniques which typically include much of the rhinal cortices. Thus, it comes as no surprise that despite efforts to select patients ideal for each of these procedures, overall seizure freedom rates have been reported as ~68–78% achieved by anterior temporal lobectomy in lesional cases (10, 11) vs. ~64% achieved by open selective amygdalohippocampectomy (12) vs. 53% seizure freedom with SLAH (5, 6). Complication rates attributed to open temporal lobectomy are also reduced with SLAH, including reduced incidence of visual field deficits (13, 14) and reduced neuropsychological deficits in naming and object recognition (15), as well as reduced hospitalization times and an improved patient experience. Nevertheless, the uniqueness of the case we report herein precludes any direct comparison to more extensive published results regarding open anterior temporal lobectomy or SLAH.

Improved patient experience with MRgLITT may also expand access to epilepsy surgery for patients fearful of open surgery. When patients are asked their opinions about epilepsy surgery beforehand, a majority (74%) expressed “anxiety of the unknown” prior to surgery, even though after surgery the vast majority (94%) reported surgery increased their independence and only a minority (19%) complained of wide-ranging psychological and neurological long-term adverse effects such as fatigue, memory, and concentration impairment (16). These anxieties don't necessarily reflect the complication rates attributable to open surgery; in the seminal study by Wiebe et al. 10% (4/40) incurred an unexpected adverse event (1 thalamic infarct causing sensory changes, 1 wound infection, and 2 declines in verbal memory interfering with occupation) and 55% (22/40) had expected non-disabling visual quadrantanopsia (17). Nevertheless, in our experience, a non-trivial fraction of patients will refuse a standard open anterior temporal lobectomy but will consider the less invasive laser ablation, even if the volume of tissue targeted for destruction is the same.

We have previously described outcomes of LITT to target lesion boundaries and networks explored by SEEG (18). In our case example, we used robotic MRgLITT to achieve an ablation comparable to anterior temporal lobectomy. An extensive treatment area was recommended based upon SEEG results which generally suggested multifocal mesial-lateral temporal lobe epilepsy. Failure to identify a particular region of rhythmic spiking and/or low voltage fast activity precluded the use of LITT to target only a small area (e.g., mesial temporal) for ablation and puts our patient at greater risk of not achieving or maintaining seizure freedom (19). Thus, our team offered extensive right temporal lobectomy, but the patient expressed an interest in avoiding a typical surgical incision and a likely visual field defect. She likewise expressed understanding that despite potential feasibility, ablation might not achieve as predictable an outcome as open surgery. The complex shape of the temporal lobe and mesial temporal structures in particular must be considered due to the limitations imposed by linear stereotactic trajectories. The curvature of the hippocampus and uncus limits the anterior medial extent of the ablation and a more lateral entry must be utilized to place the laser fiber in the uncus (20). Due to the medial curvature of the posterior hippocampal formation at the region of the lateral mesencephalic sulcus, however, the posterior body of the hippocampus could be missed. For this reason, we used two occipital trajectories to ablate the parahippocampal gyrus and rhinal cortices, the amygdala and uncus, and the head and body of the hippocampus. The remaining planum polare and inferior temporal pole and lateral temporal neocortex (including the superior temporal sulcus) were ablated using four lateral trajectories. Additional lateral trajectories were included in this particular case to assure a confluent ablation of the lateral temporal cortices, because the SEEG results implicated a widespread medial-lateral temporal epileptogenic zone. Fewer trajectories might be required to target the combination of the temporopolar and medial temporal structures alone. The combination of posterior and lateral approach trajectories is easily accommodated within a robotic workflow.

For trajectory planning, deliberate considerations must be made to (i) target the appropriate tissue for ablation and (ii) avoid key structures to minimize the risk of complications from off-target injury. In particular for the mesial temporal lobe, cranial nerves within the cavernous sinus, along the tentorium, and within the skull base are vulnerable to thermal injury, as well as the optic tracts and lateral geniculate nucleus (LGN) of the thalamus which overly the body of the hippocampus. This patient did incur an ipsilateral facial nerve injury, which resolved with conservative management by 6-months after surgery. Proximity to the skull base was considered during surgical planning, and the trajectory in this location was planned 8 mm away from the skull base to account for the expected diameter of the ablation. As the patient seemed to have no facial nerve palsy immediately after surgery but developed hemifacial weakness progressively over the first 3 days after surgery, this suggests that direct thermal injury during the ablation was not the cause. Given prior reports of prolonged blood brain barrier disruption following laser ablation including a published case of delayed optic neuritis (21), it is possible that thermal ablation may rarely precipitate autoimmune central nervous system inflammatory conditions, as in this case resembling typical Bell's palsy. Supporting this hypothesis, the facial nerve itself showed minimal changes on immediate post-operative imaging, and was found to avidly contrast-enhance on imaging obtained 3-months after surgery. Despite our attention to the location and course of the facial nerve in the skull base when ablating the basal temporal lobe, the observed nerve injury testifies to the sensitivity of this nerve to direct thermal and/or indirect inflammatory injury. We urge even greater respect for proximity to this nerve with regard to laser trajectory proximity and relative power settings. Inherent constraints of gradient echo-weighted imaging-based thermometry near bone remain a limitation of commercially available MRgLITT systems and highlight a need for further technological developments.

## Conclusion

We described the application of robotic stereotaxy and MR-guided laser interstitial thermal therapy in a combined minimally invasive surgical workflow for anterior temporal lobotomy. Robot guided MRgLITT provides a number of benefits, including those associated with minimally invasive techniques such as smaller incisions and reduced length of stay, with comparable surgical efficacy. Complications remain possible with minimally invasive techniques, and must be considered and mitigated with prior knowledge and experience. Minimally invasive techniques such as MRgLITT will not entirely replace open surgical techniques, however complement existing treatments and offer alternate therapeutic strategies in selected patients. MRgLITT may also expand access to epilepsy surgery for patients who refuse open surgery.

## Data Availability Statement

The original contributions presented in the study are included in the article, further inquiries can be directed to the corresponding author.

## Ethics Statement

The studies involving human participants were reviewed and approved by Emory University IRB. Written informed consent for participation was not required for this study in accordance with the national legislation and the institutional requirements.

## Author Contributions

KG and JW conceptualized the manuscript. KG, AD, and JW wrote the manuscript. RH and EF provided data. All authors approved the final manuscript.

## Conflict of Interest

JW: Medtronic-consulting, research contract (SLATE trial), honoraria for teaching, Neuropace-consulting, research contract, honoraria for teaching, Clearpoint Neuro/MRI Interventions-consulting, AiM Medical-consulting. EF has received research support from UCB Pharma, Xenon, Eisai, and the NINDS, and consulting fees from Biogen, Supernus, SKLife Science, and the CDC. RH is involved with the Medtronic SLATE trial but receives no financial support. The remaining authors declare that the research was conducted in the absence of any commercial or financial relationships that could be construed as a potential conflict of interest.
